# A video summarization framework based on activity attention modeling using deep features for smart campus surveillance system

**DOI:** 10.7717/peerj-cs.911

**Published:** 2022-03-25

**Authors:** Wasim Muhammad, Imran Ahmed, Jamil Ahmad, Muhammad Nawaz, Eatedal Alabdulkreem, Yazeed Ghadi

**Affiliations:** 1 Center of Excellence in Information Technology, Institute of Management Sciences (IMSciences), Peshawar, Peshawar, KPK, Pakistan; 2 Department of Computer Science, Islamia College Peshawar (Chartered University), Peshawar, Pakistan; 3 Computer Sciences Department, College of Computer and Information Sciences, Princess Nourah Bint Abdulrahman University, Riyadh, Saudi Arabia; 4 Department of Computer Science, Al Ain University, Al Ain, UAE

**Keywords:** Deep learning, Emerging technologies, Dats science, Machine learning

## Abstract

Like other business domains, digital monitoring has now become an integral part of almost every academic institution. These surveillance systems cover all the routine activities happening on the campus while producing a massive volume of video data. Selection and searching the desired video segment in such a vast video repository is highly time-consuming. Effective video summarization methods are thus needed for fast navigation and retrieval of video content. This paper introduces a keyframe extraction method to summarize academic activities to produce a short representation of the target video while preserving all the essential activities present in the original video. First, we perform fine-grain activity recognition using a realistic Campus Activities Dataset (CAD) by modeling activity attention scores using a deep CNN model. In the second phase, we use the generated attention scores for each activity category to extract significant video frames. Finally, we evaluate the inter-frame similarity index used to reduce the number of redundant frames and extract only the representative keyframes. The proposed framework is tested on different videos, and the experimental results show the performance of the proposed summarization process.

## Introduction

Academic activities are carried out on university campuses as a matter of daily routine. Video cameras are installed at various points covering all these activities and stream the video footage to a backup storage server for future correspondence. During working hours the frequency of these activities are normally high, gradually lessens after the off time and reaches its minimum level in the late night. Further, these activities do not take place on weekly holidays or other public and national holidays except those explicitly scheduled. The campus’s video surveillance system (VSS) continuously transmits video streams regardless of whether these activities are happening or not. This limits the preservation of data to short periods because a huge amount of garbage data utilizes a considerable portion of the backup storage. Further, due to the non-availability of automated content-based searching mechanisms, looking manually for a particular event or an activity in such a vast video repository is a major commitment of time and resources Choi, Chan & Yue (2016).

Video summarization (VS) has been an essential tool for many video analytic tasks, which produces a brief and concise representation of the visual content of a video. A qualitative video summary has two essential features. First, it must be representative, such that it covers all the important events of the original video, and second, it should have minimal redundancy (Ejaz, Tariq & Baik, 2012). Several VS methods have been proposed for various application domains like electronic media such as sports, TV series, movies, documentaries, *etc*., personal videos such as lifelogging, birthday parties, medical videos such as digital endoscopies, online databases such as YouTube, and many surveillance applications such as homes, traffic, agriculture, industries, public places and so on.

As far as academic campuses are concerned, a negligible amount of research work has been done in diverse and demanding areas. In this paper, we propose a deep learning-based fine-grain activities recognition and summarization method for the long video surveillance streams covering, for instance, two major academic activities Examination and Class Lecture. The proposed method will allow users to quickly navigate through large video databases and take faster decisions regarding selection, deletion, sharing, and consuming video content. The major contribution of this research work includes the introduction of a trained model for the above-stated academic activities and activity-based summarization method in a novel campus domain.

The rest of the paper is organized as follows: “Related Work” contains a review of existing video summarization techniques proposed by researchers. In “Methodology”, we explain our dataset and activity-based summarization framework. “Experimental Results and Discussion” presents our experimental results, and finally, “Conclusion” concludes our work and provides some future directions.

## Related Work

Researchers have proposed solutions for various video summarization problems in a particular application domain. However, these methods vary considerably in terms of efficiency and complexity, depending on the nature of the problem at hand.

Early methods of crucial frame selection for summary generation uses low-level feature like color, motion, and texture analysis. Obtaining color histogram (Ejaz, Tariq & Baik, 2012) for each of the successive frames of a video and selecting keyframes based on histogram difference has been one of the most commonly used approaches with the color-based methods. Similarly, optical flow analysis (Deng & Manjunath, 1997), models motion patterns to evaluate motion histograms and wavelet transforms, for texture analysis (Mahmoud, Ismail & Ghanem, 2013), are among the most popular approaches for evaluating frame saliency to generate video summaries. Instead of using a single feature, researchers also proposed unified frameworks (Gianluigi & Raimondo, 2006) which make use of multiple low-level features to generate more valuable summaries. However, the importance of these features greatly varies for different types of VS problems. To overcome these discrepancies, weighted frameworks (Ioannidis, Chasanis & Likas, 2016) were proposed, which assign weights to different features for practical saliency calculation specific to the problem under investigation. With the advancements in video technology, the use of low-level features has no longer remained an ideal approach for VS. High-level features like face detection (Lee et al., 2015), objects identification (Lin et al., 2017), emotions (Lan et al., 2016) and gestures detection (Dammak, Wali & Alimi, 2015) produces more appealing video summaries for high quality and complex video contents.

Video summarization methods greatly vary depending on the nature of the problem in various application domains and the content of the video. Clustering is one of the most extensively used techniques for summarization, where the visual contents share resemblance over different time periods in the video. Zhuang et al. (1998) authors have proposed a VS method by grouping video frames that potentially have high similarity, calculating cluster center in each group, and finally combining all the cluster centers to generate a static video summary. Several approaches such as Partitioned Clustering (Valdés & Martnez, 2007), Spectral Clustering (Damnjanovic et al., 2008; Stefanidis et al., 2000) and K-Mean clustering (Amiri & Fathy, 2010) have been used for different summarization problems. Many techniques such as High-Density Peak Search (Wu et al., 2017), Self-Organizing Maps, and Gaussian Mean (John, Nair & Kumar, 2017) are proposed by researchers to improve the keyframe selection process further to produce less redundant and qualitative video summaries.

Professional videos normally have a hierarchical structure and consist of a finite number of scenes where a scene is a collection of multiple shots. Shot boundary detection in such videos is a complex task. Transformation Co-efficient distance (Girgensohn & Foote, 1999), Frame packing algorithm (Uchihachi, Foote & Wilcox, 2003), Hierarchical clustering using Color Histogram distance (Chheng, 2007) and SIFT point distribution histogram (Hannane et al., 2016) are among the most commonly used techniques proposed by different researchers for detecting shot boundaries. Salient frames from each shot are combined to obtain a summary of the full-length video. As opposed to professional videos, the contents of surveillance videos are highly unstructured, normally having a static background and involve very less or even no camera motion. In such situations, the most desirable aspect of performing the intended VS is the detection of the behavior of the moving objects. Qiu et al. (2008), and Stefanidis et al. (2000) authors extract and use trajectory information of the moving nodes using Spatio-temporal features for the generation of video summary. Similarly, Curve Simplification based summarization (Bulut & Capin, 2007; Song et al., 2016) evaluate frame saliency by analyzing motion behaviors more efficiently in a situation where camera motion is involved in surveillance videos.

Apart from normal video summarization, which focuses on the evaluation of individual frame saliency, the detection of events of interest has been a challenging task in event-based video summarization (Wang & Ngo, 2011). Damnjanovic et al. (2008) authors use a reference frame representing an activity to identify frames with identical activity by calculating frame difference, assigning weights to each different frame, and finally selecting high weight frames as part of the summary. A Context-aware video summarization (Zhang, Zhu & Roy-Chowdhury, 2016) process performs Spatio-temporal correlation among events and uses this information for summary generation in surveillance videos. Another event detection method (Zhang et al., 2017) where the proposed algorithm identifies a common event along with multiple videos and performs saliency calculation in an unsupervised manner. The performance of event-based summarization significantly depends on selecting and extracting appropriate features for classification. Rather than using hand-crafted features, researchers recommend learned features because Convolution Neural Network (CNN) inherits distinctive learning ability for classification of video contents such as complex activity classification tasks (Karpathy et al., 2014; Rodrguez-Moreno et al., 2019).

Poleg et al. (2016), Alom et al. (2017), and Ji et al. (2012) achieved state-of-the-art results while using CNN features for the proposed activity detection problems. Due to its remarkable results, many deep learning based frameworks (Ahmed et al., 2020a; Ahmad, Ahmed & Jeon, 2021; Ahmad et al., 2020, 2020b; Mahasseni, Lam & Todorovic, 2017; Plummer, Brown & Lazebnik, 2017) are proposed by the researchers for video summarization in various application domains. Authors performed summarization of surveillance videos (Muhammad, Hussain & Baik, 2020), egocentric videos (Jain, Rameshan & Nigam, 2017; Del Molino et al., 2016) and industrial videos (Muhammad et al., 2019) using deep features. Similarly, Koutras, Zlatinsi & Maragos (2018) presented an event based method using a combination of 3D-CNN and 2D-CNN architectures, a unified framework, for high quality structured videos. Deep learning based methods (Ahmed et al., 2021b; Ahmed, Anisetti & Jeon, 2021c; Ahmed et al., 2021d; Ahmed & Jeon, 2021; Ahmed et al., 2021a; Ahmed et al., 2022), is also used by researcher such as Wasim et al. (2021) presented a novel deep learning based automated system for academic activities recognition.

Researchers have introduced many state-of-art activity recognition methods for various application domains with the availability of several activity data sets. Unfortunately, none of these data sets or methods have been developed explicitly for academia and do not cover academic activities. Thus, an automated deep learning-based academic activities recognition system is introduced in this work.

## Methodology

**Figure 1:**
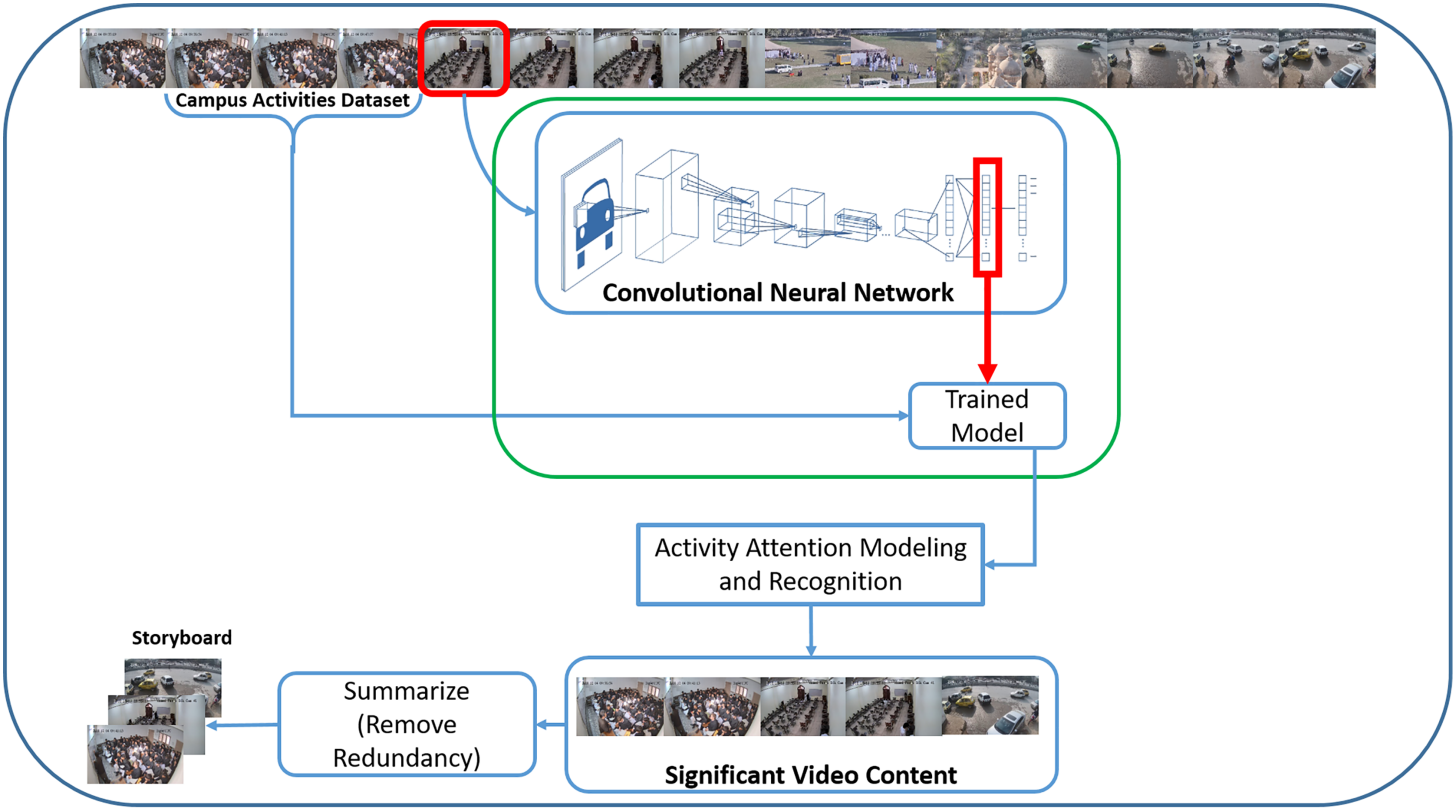
CNN based campus surveillance video summarization framework.

Our proposed Campus Surveillance Video Summarization (CSVS) method consists of five main tasks, as shown in Fig. 1. The first one is the development of a realistic academic activities dataset, because the existing datasets (Fan, Wang & Huang, 2016; Singh, Velastin & Ragheb, 2010; Denina et al., 2011; Leyva, Sanchez & Li, 2017; Wang et al., 2014; Malon et al., 2018; Awad et al., 2021) researchers have been using to solve various summarization problems, lack academic activities. Second, training a CNN model with a novel set of academic activities. The third step performs activity recognition using activity attention scores generated by the trained model. In the next phase, saliency evaluation and significant activity frame extraction are performed to obtain a coarse-grained video summary based on activity attention score. Finally, the removal of redundant frames and extraction of keyframes is carried out to generate the final summary.

### Data set

The video data required for this research work is obtained from the surveillance repository of Islamia College Peshawar (ICP), a public sector university situated in Khyber Pakhtunkhwa, providing higher education under the governance of Higher Education Commission (HEC), Pakistan. The ICP smart campus (https://icp.edu.pk/page.php?abc=201905090555484) surveillance network consisting of 771-night vision cameras, installed at all the sensitive areas, including the classrooms, capture, and streams all the routine activities to a 92 Terabyte data server.

For instance, our proposed Campus Activities Dataset (CAD) covers two main academic activities that generally happen within a classroom, Class and Examination (Exam). Class refers to an ongoing teaching session, while Exam is some written assessment conducted from the students. Both these activities are further categorized into a total of 14 fine-grain activities. The Class is divided into six categories: Writing on Board, Explanation (Teacher explaining a topic), Board Cleaning, Class Entry, Class Exit, and Class Break. Entry and Exit refer to somebody entering or leaving the classroom while Class Break is the recess period when students are informally sitting in the classroom during an off session. Similarly, Exam is divided into eight categories which are Paper Distribution, Paper Attempt (students writing their answers), Paper Signature (Invigilator signing the answer book), Attendance (Ensure student presence during exam), Paper Collection, Exam Entry, Exam Exit, and Exam Break. This categorization aims to cover the whole activity for the proposed activity recognition and summarization task. The dataset development is done in the following steps.

- Sufficient video streams are collected from the surveillance data server. These videos are then scrutinized to select appropriate streams based on different camera angles, camera location, visual clarity, and the required contents.
- For each of these 14 activities, appropriate video segments are manually cropped from the selected video streams. The video segments are variable in length depending on the duration of a particular activity in the video. The number of video segments in each category is shown in Table 1.
- The video segments are converted into video frames for more precision. All the irrelevant, noisy, and redundant frames are removed from each category, and we are left with only those frames that best represent the target activity, Table 2.
- Finally, the frames are labeled in sequential order as they appear in the original video segment.

**Table 1:** Characteristics of campus activities dataset.

S. No.	Category	No. of video samples	Duration (H:M:S)	No. of frames
1	Writing on Board (WB)	104	00:28:52	14,507
2	Explanation (Exp)	161	00:39:27	15,052
3	Board Cleaning (BC)	63	00:16:11	9,237
4	Class Entry (CE)	136	00:19:03	15,271
5	Class Exit (CEx)	96	00:13:09	11,918
6	Class Break (CB)	42	00:09:36	6,634
7	Paper Distribution (PD)	86	00:21:43	14,194
8	Paper Attempt (PA)	121	00:18:50	12,871
9	Paper Signature (PS)	87	00:22:49	15,227
10	Attendance (Att)	109	00:23:36	11,829
11	Paper Collection (PC)	207	00:26:19	16,102
12	Exam Entry (EE)	121	00:15:17	15,363
13	Exam Exit (EEx)	131	00:15:59	13,488
14	Exam Break (EB)	52	00:11:42	6,357

**Table 2:** Architecture of the CNN model.

Layer	Kernels	Size	Stride	Activation
Conv – 1	128	(3,3)	(1,1)	Relu
Max-Pooling	–	(2,2)	(2,2)	–
Conv – 2	64	(3,3)	(1,1)	Relu
Max-Pooling	–	(2,2)	(2,2)	–
Conv – 3	32	(3,3)	(1,1)	Relu
Max-Pooling	–	(2,2)	(2,2)	–
Conv – 4	16	(3,3)	(1,1)	Relu
Max-Pooling	–	(2,2)	(2,2)	–
FC-01	–	500	–	–
FC-02	–	1000	–	–
Output	–	14	–	Softmax

### Model training

As discussed in “Related Work”, CNN models possess significant learning ability and have a high accuracy rate in activity classification on large datasets. Several CNN models (Dhillon & Verma, 2020) exist that the researchers have been using for various problems. While using a pre-trained CNN model like AlexNet or VGG-16, having a lengthy architectural pipeline and a large number of kernels, for the proposed problem would encounter additional computational and processing complexity because there is significantly less variation among the video frames in the CAD dataset, the cameras are fixed, and no camera motion is involved. Hence, we use a customized model which consists of 04 convolution layers, 04 max-pooling layers, 02 fully-connected (FC) layers, and 01 classification layer in such a way that a max-pool layer follows each convolution layer. Next to the last max-pool, two FC layers are followed by a Softmax classification layer, as shown in Table 2.

Before starting the training process, the CAD dataset is initially split into Training, and Test sets where the Training set contains 90% and the Test set contains 10% of the total frames in each category. The training data is further divided into Train and Validation sets in such a way that each set comprises 70% and 30% frames, respectively. The video frames (VF) are resized to dimensions (224 × 224) having 03 color channels. The input layer read each video frame and the convolution function, Eq. (1), produces an output feature map of size (*h*1, *w*1, *c*2), Eq. (2), by applying a filter kernel K of size (*k*, *k*, *c*) to each VF with dimensions (*h*, *w*, *c*).

(1) $$Convolve\left( {VF,K} \right)(x,y) = \sum\limits_{\left( {i = 1} \right)}^h {\sum\limits_{\left( {j = 1} \right)}^w {\sum\limits_{\left( {k = 1} \right)}^c {{K_{\left( {i,j,k} \right)}}} } } V{F_{\left( {x + i - 1,y + j - 1,k} \right)}}$$

(2) $${F_{\left( {x,y} \right)}}\left( {Convolve\left( {VF,K} \right)} \right) = \left( {{{h + 2P - k} \over S} + 1} \right),\left( {{{w + 2P - k} \over S} + 1} \right)\ for\ S \gt 0$$

or

(3) $${F_{\left( {x,y} \right)}} = \left( {h + 2P - k,w + 2P - k} \right)\ for\ S = 0$$

where,

*h* = height of video frame

*w* = width of the video frame

*c* = no. of color channels

*k* = size the kernel

*P* = Padding size, and

*S* = Stride

The max-pool layer reduces the number of parameters received from the convolution layer by keeping only the high-level features representation and thus avoiding computation of passive weights, which ultimately helps reduce the chances of over-fitting. This operation is carried out by applying a function, Eq. (3), which only reduces the dimensions of the frame *F*_(*x*,*y*)_ while keeping the number of channels, *c*, unchanged.

(4) $$x,y(Pool(F_{(x,y)})) = {{\left( {h + 2P - k} \right)} \over {S + 1}},{{\left( {w + 2P - k} \right)} \over {S + 1}},c)\ for \ S \gt 0$$

or

(5) $$x,y(Pool(F_{(x,y)})) = \left( {h + 2P - k,w + 2P - k,c} \right)\ for\ S = 0$$

The feature weights, *f*_*w*_, are converted to a one-dimensional vector by a flatten layer, passes it to the fully connected layer, which generates another vector of learned parameters *z*_*j*_, Eq. (4), and mapping each parameter to every node *j* of the *i*th FC layer.

(6) $$z_j^{[ i ]} = \sum\limits_{( {l = 1} )}^{{n_{i - 1}}} f {w_j},{l^{[ i ]}},a_l^{[ {i - 1} ]} + b_j^{[ i]} \to a_j^{[ i ]} = {\phi ^{[ i ]}}\left( {z_j^{[ i ]}} \right)$$

where, $a_l^{\left[ {i - 1} \right]}$ is the result of the last pool layer and $b_j^{\left[ i \right]}$ is the bias. Finally, a total of *n*_*l*−1_ × *n*_*l*_ learned weights *w*_*j*,*l*_ at *l*th layer are obtained.

### Activity recognition and attention modeling

In this step, we use our trained model for the proposed activity recognition task. Initially, the target video *Vt* is first converted into a set of *NF* frames, Eq. (5); then, features are extracted for each of the input frames using Eq. (2) and stored as a one-dimensional vector *FW*.

(7) $${V_t} = \left\{ {F\left( {t + i} \right)|i = 0,1,2 \ldots {n_{NF - 1}}} \right\}$$

The activity attention function, Eq. (6), calculates attention score in the range (0, 1) by obtaining exponential value for each element of *FW* and dividing it with the sum of all values. The output layer, softmax, is configured with 14 categories, and for each of the output categories, an attention score is calculated for every individual frame in the video sequence, *i.e*., a 1 × 14 vector is maintained for every video frame. Thus we obtain a total of NF vectors of the size 1 × 14 for all the frames of *V*_*t*_ such that each (1, *i*)th value, for *i* = 1, 2,…,14, corresponds to one particular output category.

(8) $$S\left( {FW} \right)i = {{{e^{F{W_i}}}} \over {\sum\nolimits_{j = 1}^k {{e^{F{W_j}}}} }}\ for\ i = 1\ K\ and\ FW = \left( {{z_1}{z_k}} \right)$$

The activity recognition process is carried out by extracting activity frames based on their generated attention scores for the corresponding activity category. The decision that what activity is going within the input video frame is indicated by the highest attention value associated with a particular category.

### Saliency evaluation and summary generation

The attention score, representing the confidence level of each of the activity frames, may be different from others depending on the content of the video frame. For summary generation, we extract the salient frames containing significant visual content. An attention score of 1, *i.e*., 100% match, associated with any activity frame is considered a significant frame, and all other frames with lower values will be dropped. The obtained sequence of frames belonging to the same activity possesses a high level of inter-frame similarity. We remove all such redundant frames and extract only the keyframes (KF) with a high content variation. We apply the Mean Square Deviation (MSD) function for keyframe extraction, which returns a difference value of the two activity frames. For any two frames, F1 and F2, with dimensions (*m* × *n*), the difference value is evaluated as follows.

(9) $$DiffVal = {1 \over {m \times n}}\sum\limits_{i = 0}^{m - 1} {\sum\limits_{j = 0}^{n - 1} {{{\left[ {F1\left( {i,j} \right),F2\left( {i,j} \right)} \right]}^2}} }$$

Using Eq. (7), difference values are evaluated for the whole frame sequence, and a one-dimensional vector is maintained where each index location contains the difference value of the two consecutive frames. In this way, a total of n difference values are obtained for the *N* number of frames. A threshold value is set, Eq. (8), whereas any frame’s difference value exceeding this threshold will be considered a keyframe and become part of the final summary.

(10) $$Thresh = {1 \over n}\sum\limits_{i = 1}^n D iffVal$$

The threshold value is inversely proportional to the number of keyframes and can be set, high or low, to obtain the desired number of keyframes. Finally, the summary is generated by combining the keyframes into a storyboard *V*_*sum*_ as shown in Eq. (9)

(11) $${V_s}um = \sum\limits_{i = 0}^{{n_K}F} {{F_{KF}}} \left( {t + i} \right)$$

## Experimental Results and Discussion

All the experiments are performed on a machine with Intel Core-i5 3.2 GHz CPU, 24 GB RAM, and a Graphics Processing Unit (GPU) NVidia GeForce GTX 1080 Ti. The programming environment is Python with Keras, Numpy, Tensorflow, and other libraries for visualization and performance monitoring such as Scikit-Learn, OpenCV, *etc*. The programming environment is configured on Windows 10 operating system.

**Figure 2:**
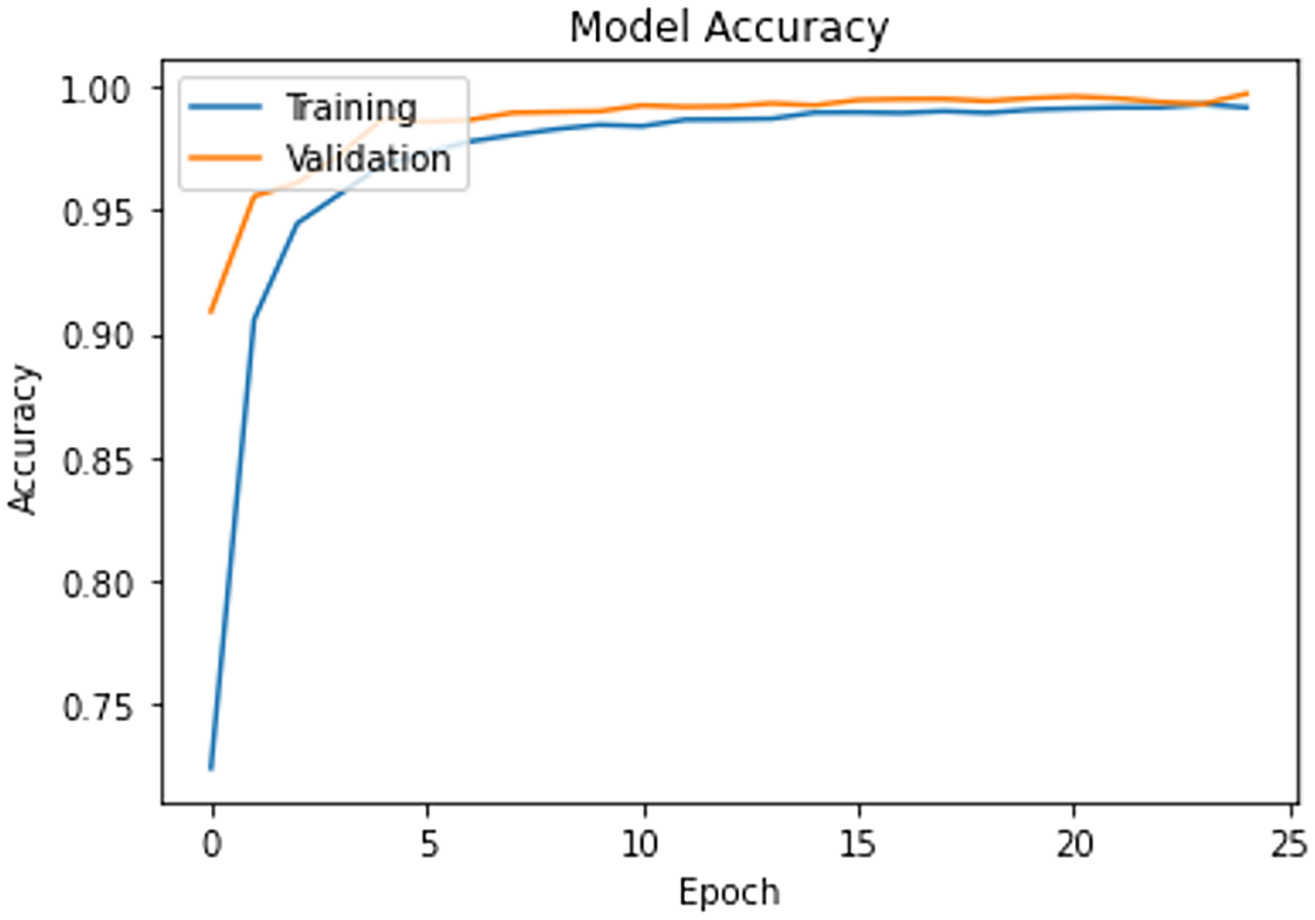
Training progress and train-validation accuracy.

The model is trained on the CAD dataset with 14 categories. The training process is set for 25 epochs with a learning rate of 0.01 and a batch size of 32. Adam optimizer is used to handle noisy content as surveillance data are most vulnerable to various noises. The validation data is used to check the model’s learning progress during the training process. Figure 2 shows the progress of the training process, and it is clear that the model presents smooth generalization on our dataset while achieving almost 98% Training-Validation accuracy.

**Figure 3:**
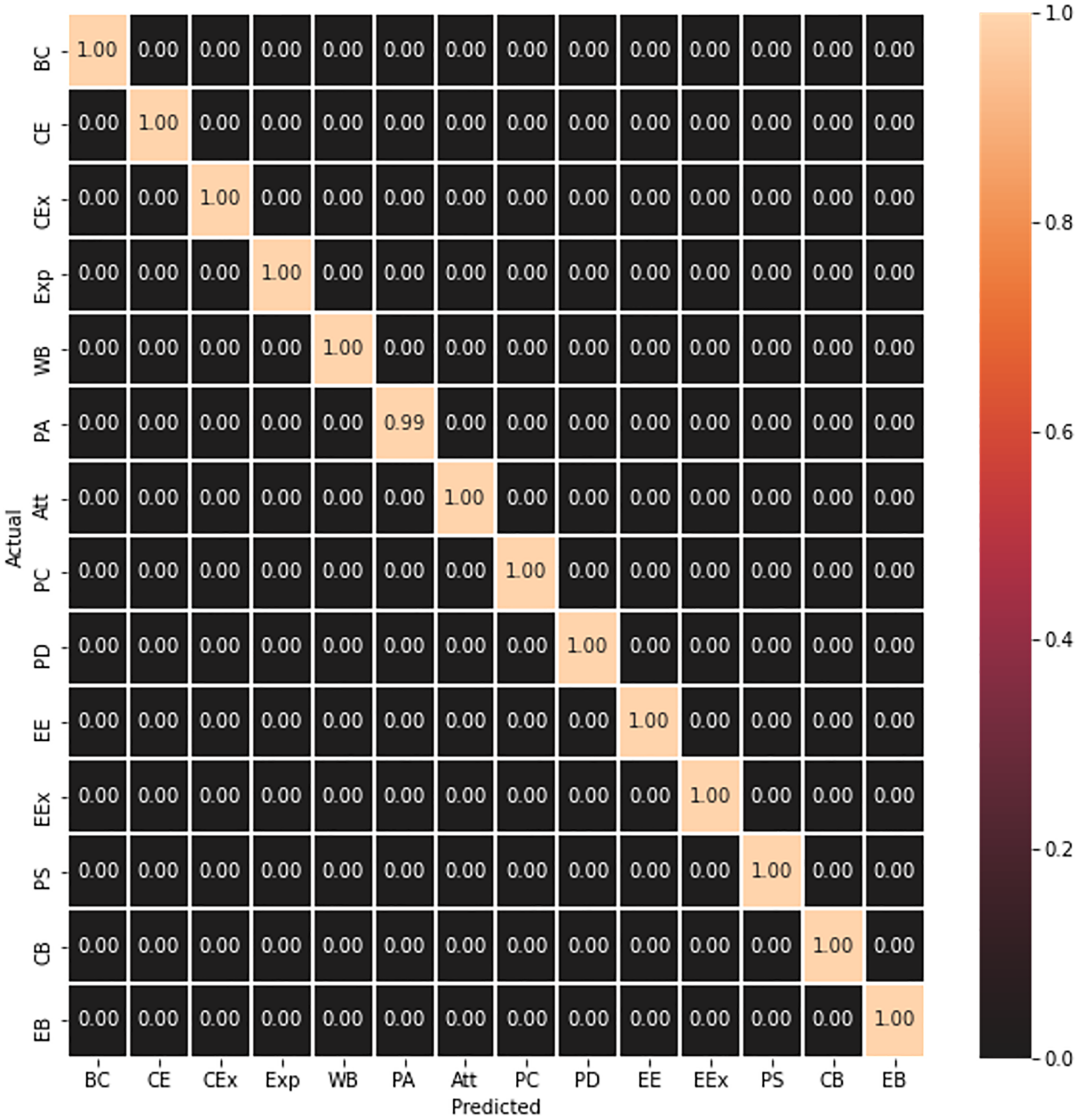
Concept matrix representing prediction result of test frames.

The trained model is further evaluated on the test data, which was 10% of the CAD dataset as discussed in “Model Training”, and it can be seen in Fig. 3 that it has correctly predicted all the test frames.

**Figure 4:**
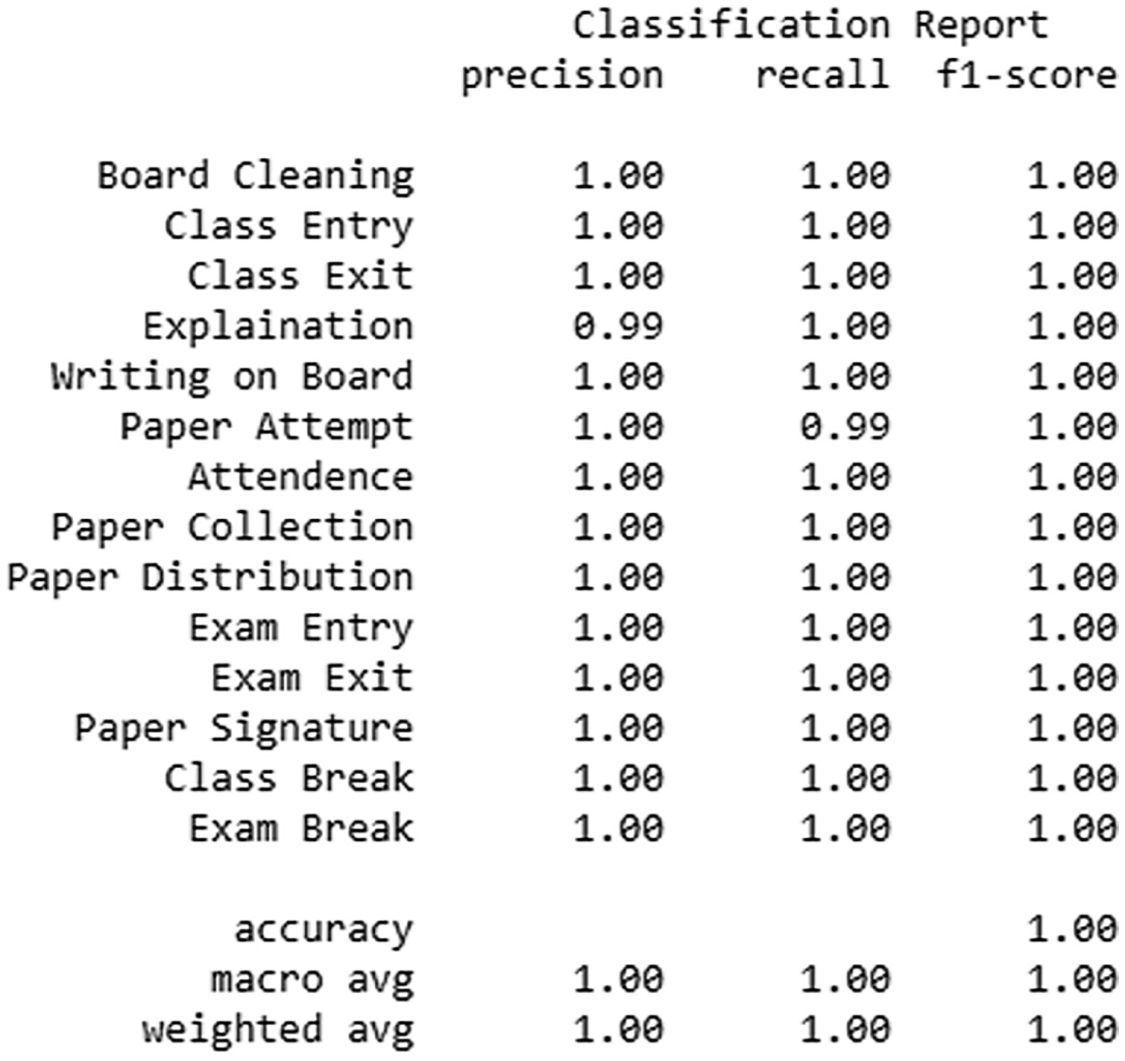
Various classification metrics showing the classification performance of the trained model.

The accuracy and various other classification metrics like Precision, Recall and F-Measure confirm the efficiency of our model and are depicted in Fig. 4.

We have obtained fresh videos from our surveillance repository for activity recognition and cropped appropriate segments from these videos. The video segments were then integrated into a single video in such a way that each video was representative with respect to the diversity of contents, camera angles, and camera location. The proposed framework was tested with four (04) videos, Video 1, Video 2, Video 3, and Video 4. Video 1 includes the set of all activities that our model was supposed to recognize. Video 2 contains a sequence of video segments that were taken from examination videos, and Video 3 is a collection of teaching video segments obtained from classroom lecture videos. In addition, however, manually recorded selective academic video segments from YouTube videos were combined into Video 4.

**Figure 5:**
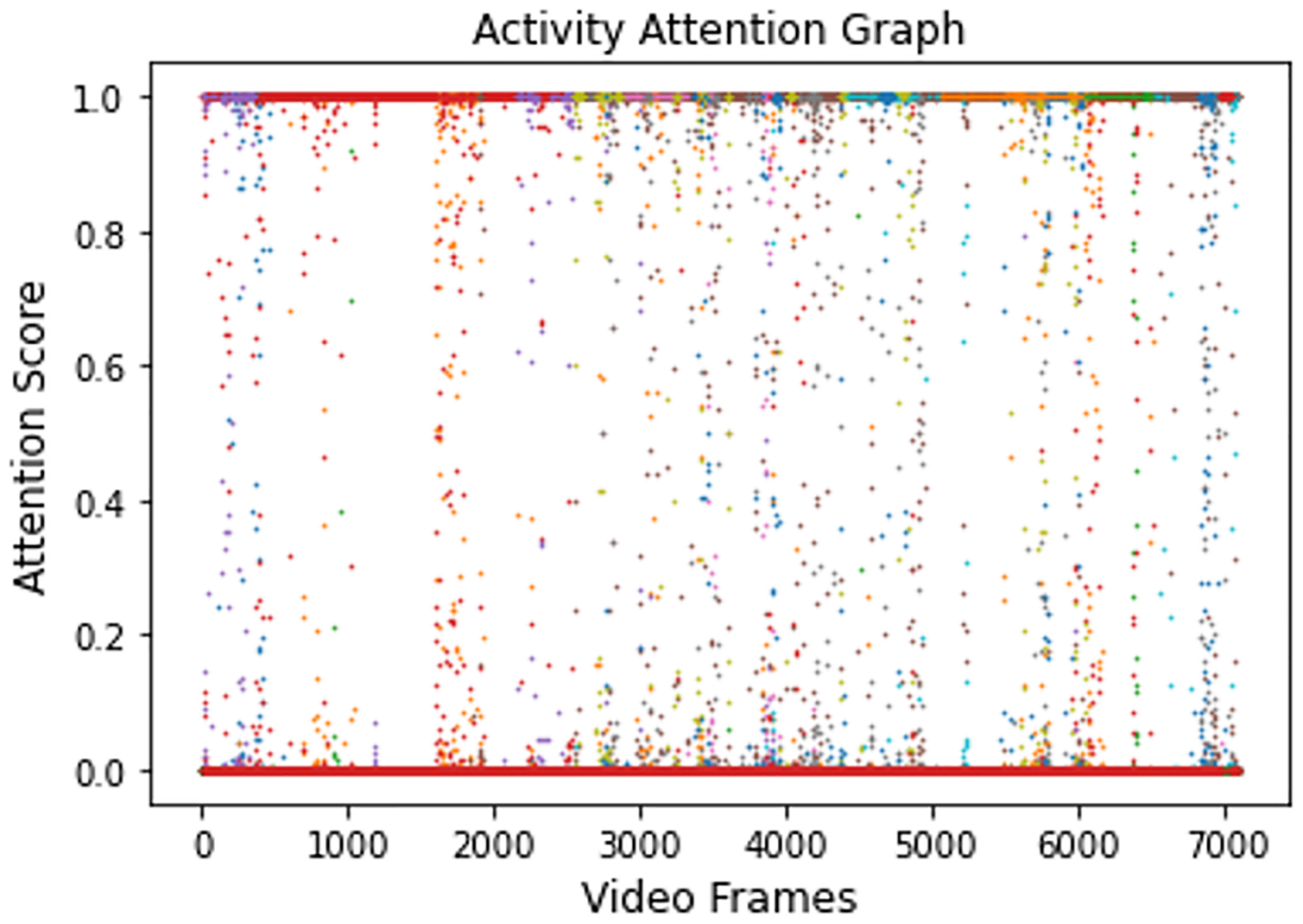
Activity attention plot showing all attention values of Video 1.

Video 1 is supplied to the trained model for evaluation. For each frame of this video, the model has successfully generated an attention score. Figure 5 shows the attention scores generated for all of the activities present in Video 1.

**Figure 6:**
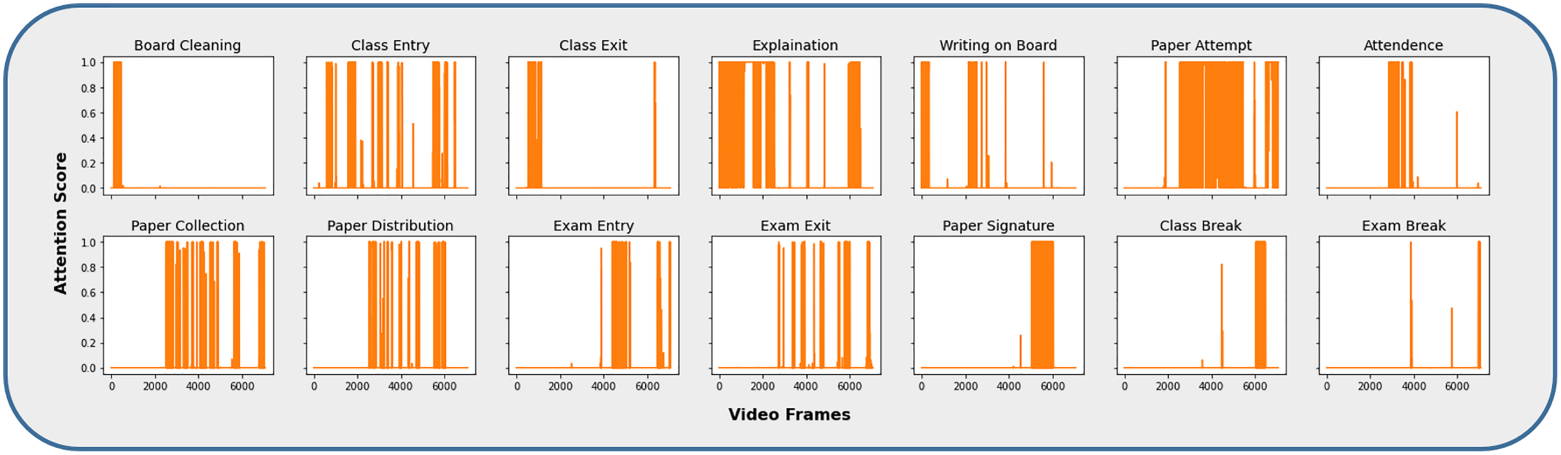
Video frames corresponding to individual activity category.

The video frames representing different activities, such as the CAD dataset, were extracted by obtaining the attention scores associated with each category, as shown in Fig. 6. The majority of the activity frames have higher attention values and are significantly closer to the highest value, *i.e*., 1. It is because these activities are parts of one of the main activities, *i.e*., Class Lecture or Exam. For example, during an ongoing lecture session, a teacher explains a topic, writes on the board, an entry or exit to or from the classroom happens during the same session. Therefore, the activities are differentiated on the basis of the highest match, maximum attention value, for a particular category. Similarly, the same process has been adopted for activities that belong to the examination.

**Figure 7:**
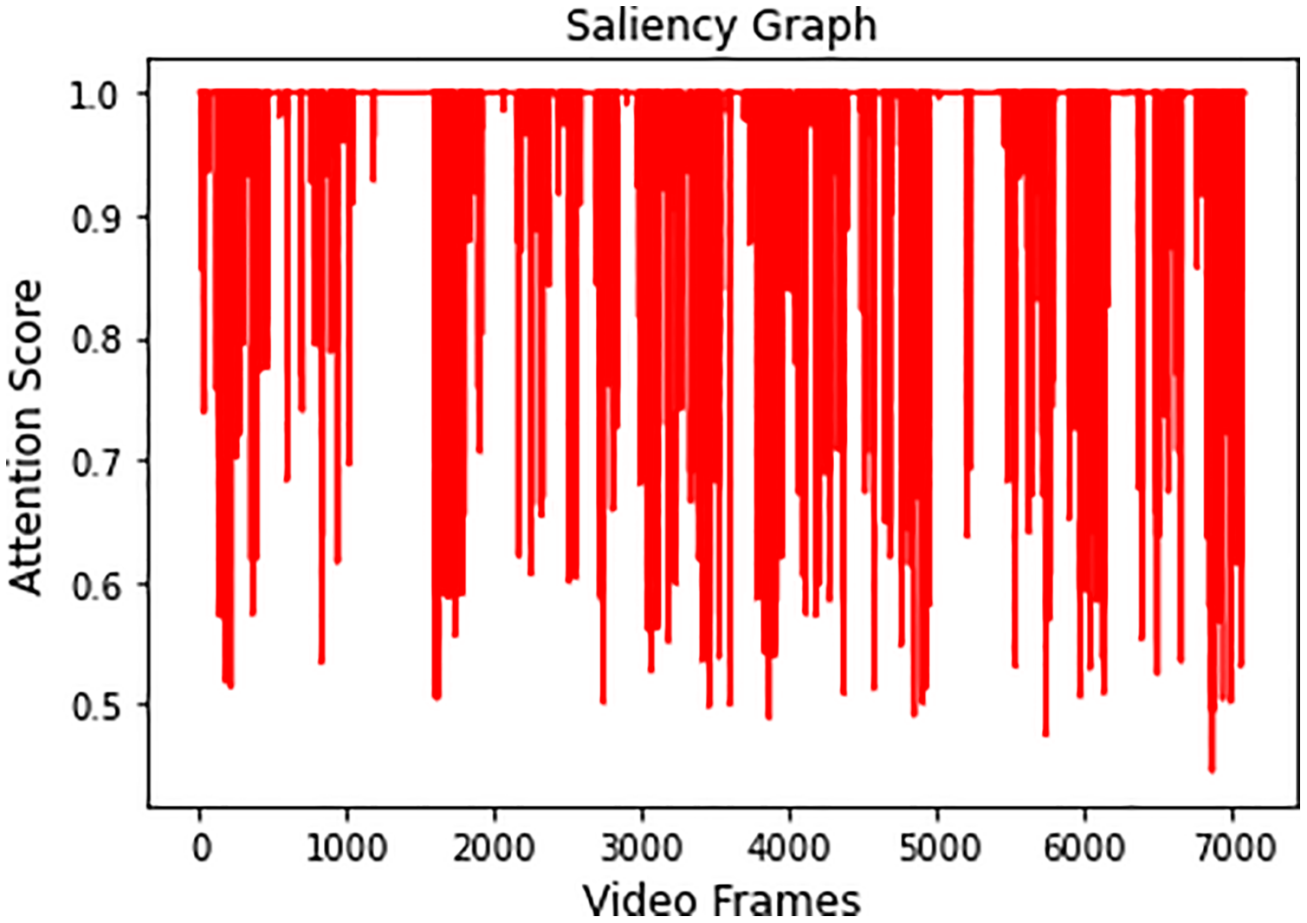
Graph showing salient activity frames from all the recognized activities.

Video 1 is summarized by obtaining all the salient frames from all categories. This is achieved by obtaining the highest attention value associated with each frame. Figure 7 shows the saliency graph of Video 1.

**Figure 8:**
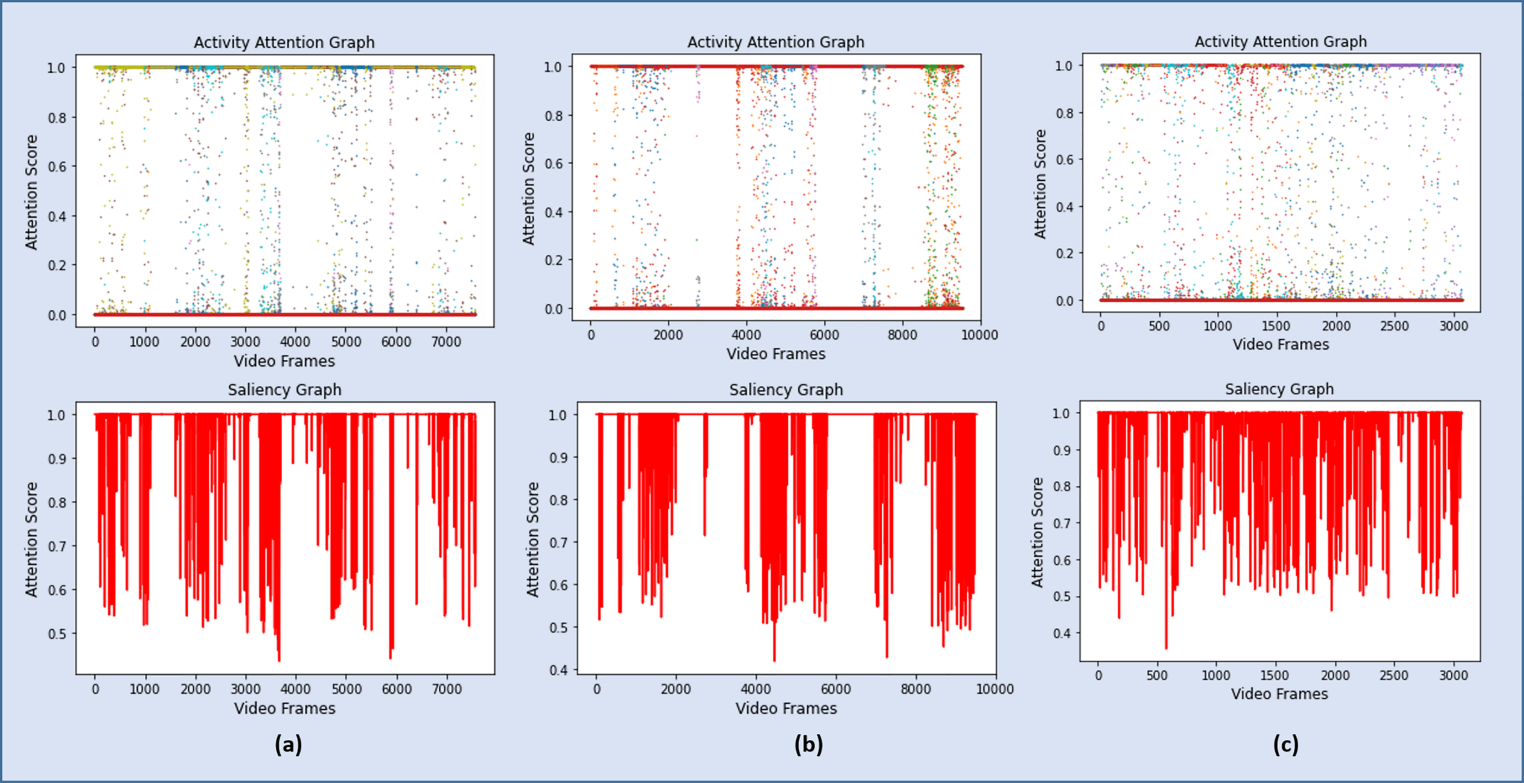
Graphs showing the attention scores and the corresponding salient frames for Video 2, Video 3 and Video 4.

As discussed in Section 3.4, a sequence of 6,660 significant frames was extracted from a total of 7,078 video frames with a percentage accuracy, measured as a ratio of significant frames to the total number of video frames of 94%. The redundant frames were removed from the sequence of significant frames by applying Eq. (7), and the final storyboard contains a total of 295 keyframes, based on Eq. (5), which is approximately 4% of the original video containing representative frames from all the categories. The same process is repeated for Video 2, Video 3, and Video 4, where the Activity Attention and Saliency Graph of these videos are shown in Figs. 8A–8C, respectively.

**Figure 9:**
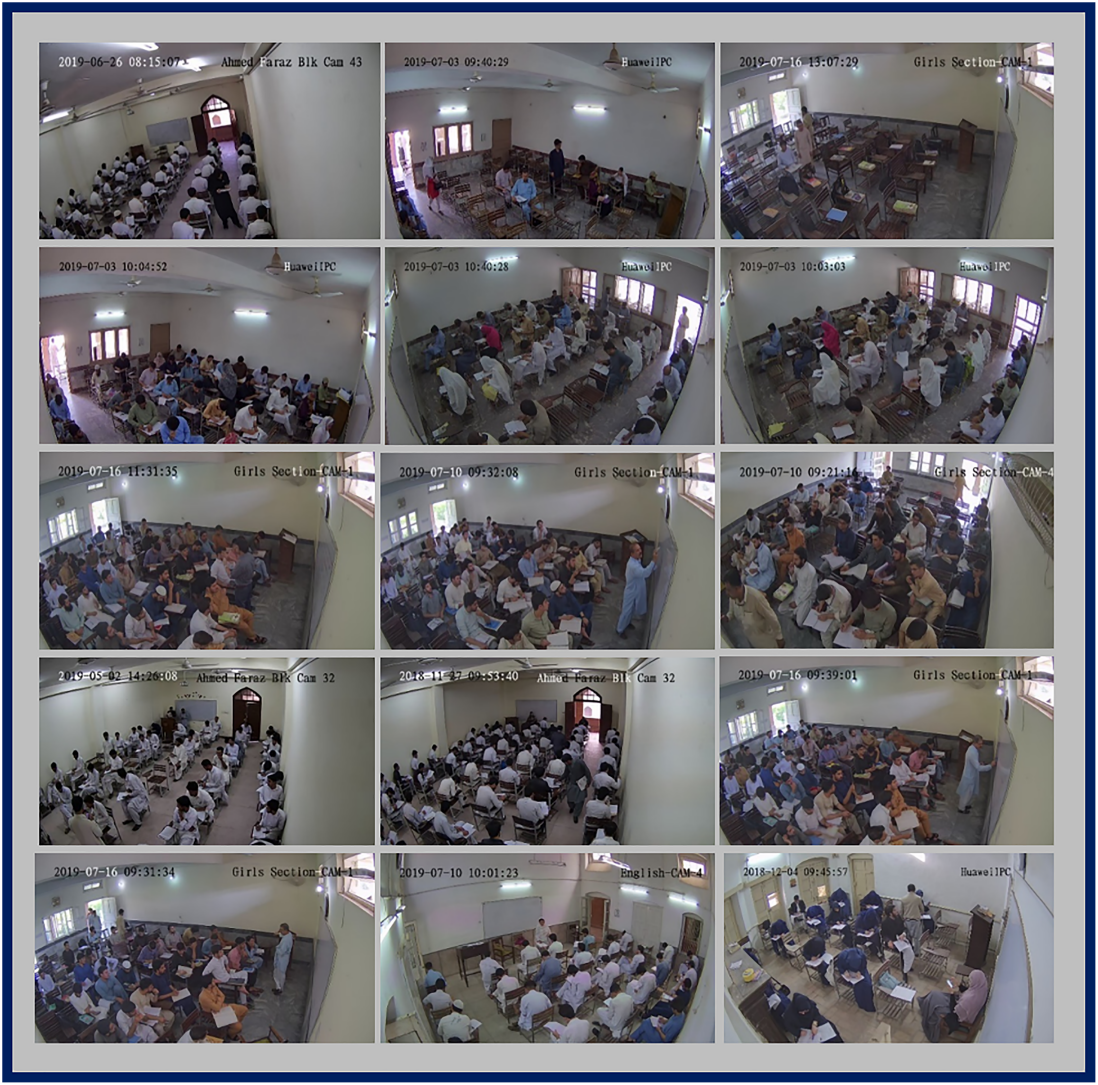
A representative key frame from each activity category in Video 1 story board.

**Figure 10:**
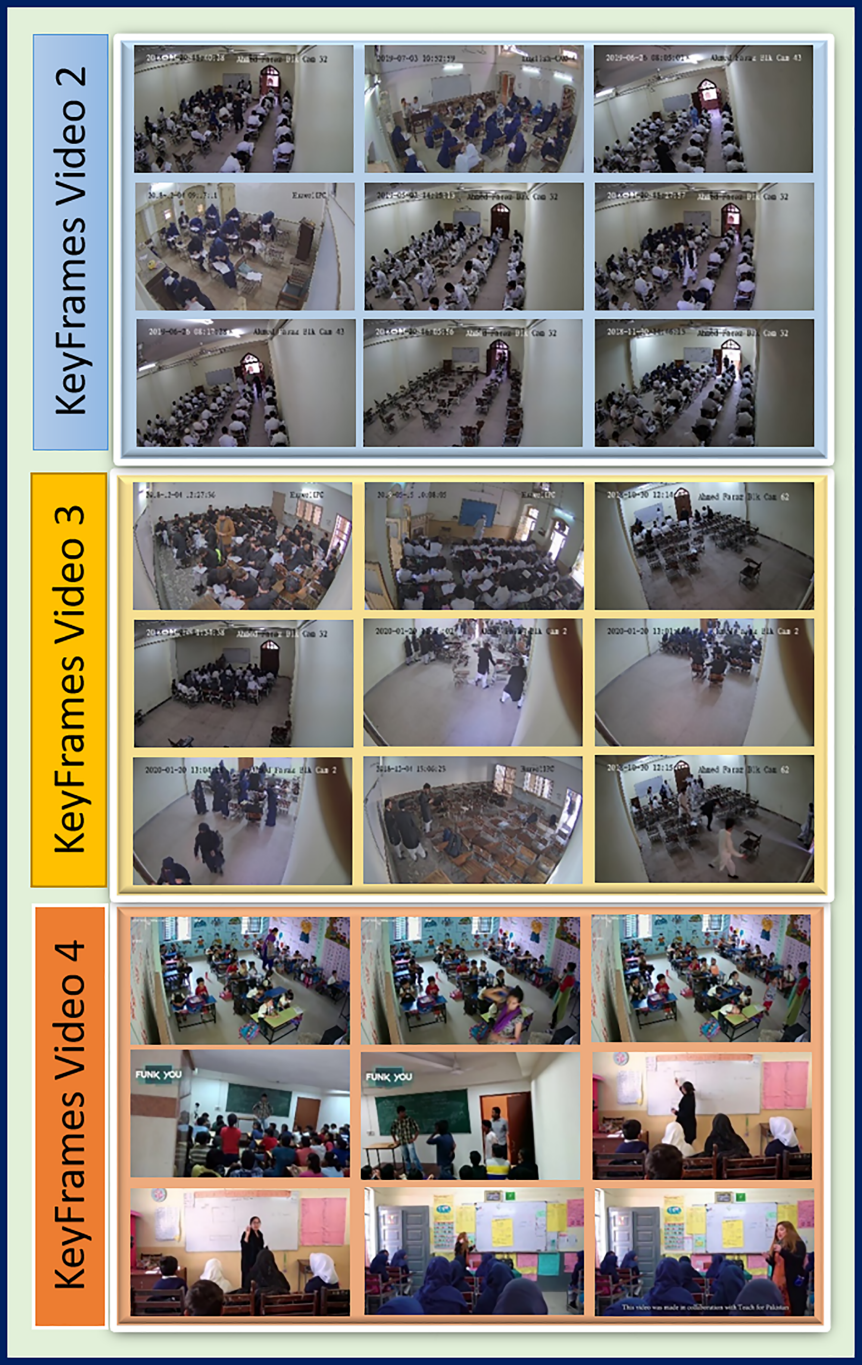
Sample key frames extracted from Video 2, Video 3 and Video 4.

The performance of the proposed framework on the four test videos has been summarized in Table 3. A quantitative summary of these four test videos is presented in Table 3. It clearly shows that for surveillance videos, Video 1, Video 2, and Video 3, the accuracy of the proposed framework insignificant activity detection is more than 94% while producing a concise summary, 4%, 1% and 3.8% respectively, of the original video. On the other hand, the model has identified only 66% of the activity frames in Video 4. It is because these videos segments are highly structured and recorded with high-resolution cameras with a limited focus in a completely different environment. However, the summarization module has efficiently produced a short summary of the identified activities covering almost all of the significant activities. Some of the representative keyframes from Video 1 are shown in Fig. 9, while Fig. 10 contains a few keyframes from the rest of the video summaries.

**Table 3:** Performance of the proposed summarization process on test videos.

Test video	Total frames	Significant frames	Detection accuracy (%)	Key frames	Size of video (%)
Video 1	7,078	6,660	94.1	295	4
Video 2	7,570	7,193	95.0	82	1
Video 3	9,539	8,991	94.2	363	3.8
Video 4	3,070	2,031	66.1	215	7

The keyframes were obtained by using an average threshold value, using Eq. (8), but can be set to a higher or a lower value to obtain the desired number of keyframes. One limitation associated with threshold setting is that if a significantly higher value is set, a much shorter summary can be obtained, but, in this case, one may miss many important activities and hence may compromise the information covered in the final storyboard. Similarly, a smaller value will increase the size of the final summary by the inclusion of a large number of redundant frames.

## Conclusion

The proposed framework is mainly focused on the practical use of the video summarization process in a novel campus domain. The results state that the proposed method is producing short summaries of the target academic activities captured by the campus surveillance system. The short representation of long video streams is of significant importance because the generated summary presents a glimpse of all the important activities in a short period. Furthermore, it will allow the administrators and other stakeholders to make a faster decision regarding the selection of desired content in an efficient manner by reducing the time required in the overall navigation and video retrieval process.

One of the main hurdles in this research domain is that organizations are reluctant to provide video data due to certain constraints regarding data privacy which amounts to the scarcity of research focus in such a demanding application domain. This piece of research effort is a step toward opening ways for quality research in this area. The video analytic researcher shall primarily focus on proposing generalized trained models and unfold challenges for similar problems. We further aim to propose frameworks for generating personalized video summaries using Long Short Term Memory (LSTM) and 3D-CNN architecture.

## Supplemental Information

10.7717/peerj-cs.911/supp-1 Supplemental Information 1 Source code used in the project.

10.7717/peerj-cs.911/supp-2 Supplemental Information 2 Data Set used in the project.

## Additional Information and Declarations

### Data Availability

The following information was supplied regarding data availability: The code and data are available in the Supplemental Files.
