# Supplementary figures and images for: A video summarization framework based on activity attention modeling using deep features for smart campus surveillance system
Source: PeerJ Comput Sci. 2022 Mar 25;8:e911. doi: 10.7717/peerj-cs.911 (PMC9044333; doi:10.7717/peerj-cs.911)

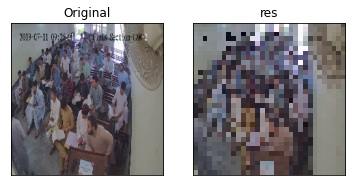

Supplement: Supplemental Information 2 [file peerj-cs-08-911-s002.zip › DataSet/1 - Copy.png]

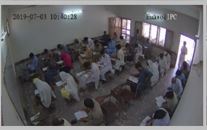

Supplement: Supplemental Information 2 [file peerj-cs-08-911-s002.zip › DataSet/10 - Copy.JPG]

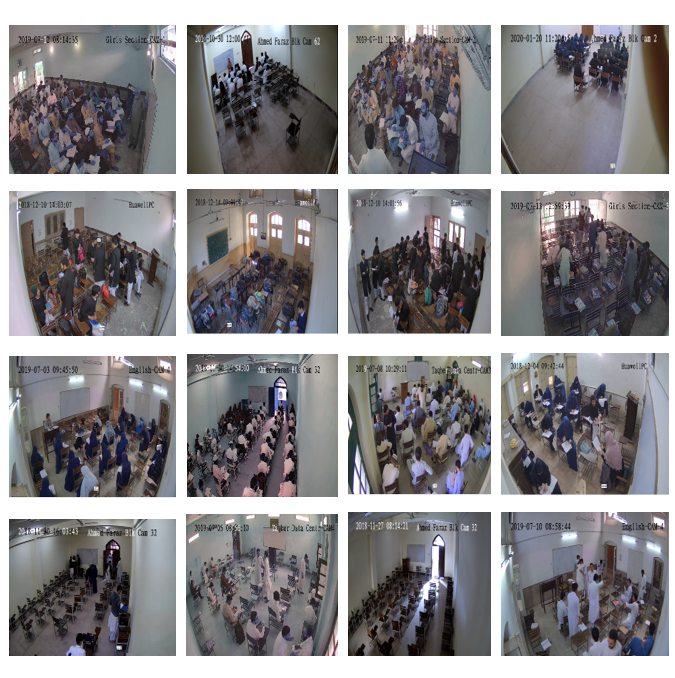

Supplement: Supplemental Information 2 [file peerj-cs-08-911-s002.zip › DataSet/11 - Copy.png]

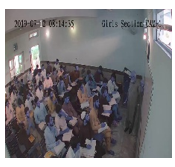

Supplement: Supplemental Information 2 [file peerj-cs-08-911-s002.zip › DataSet/12 - Copy.png]

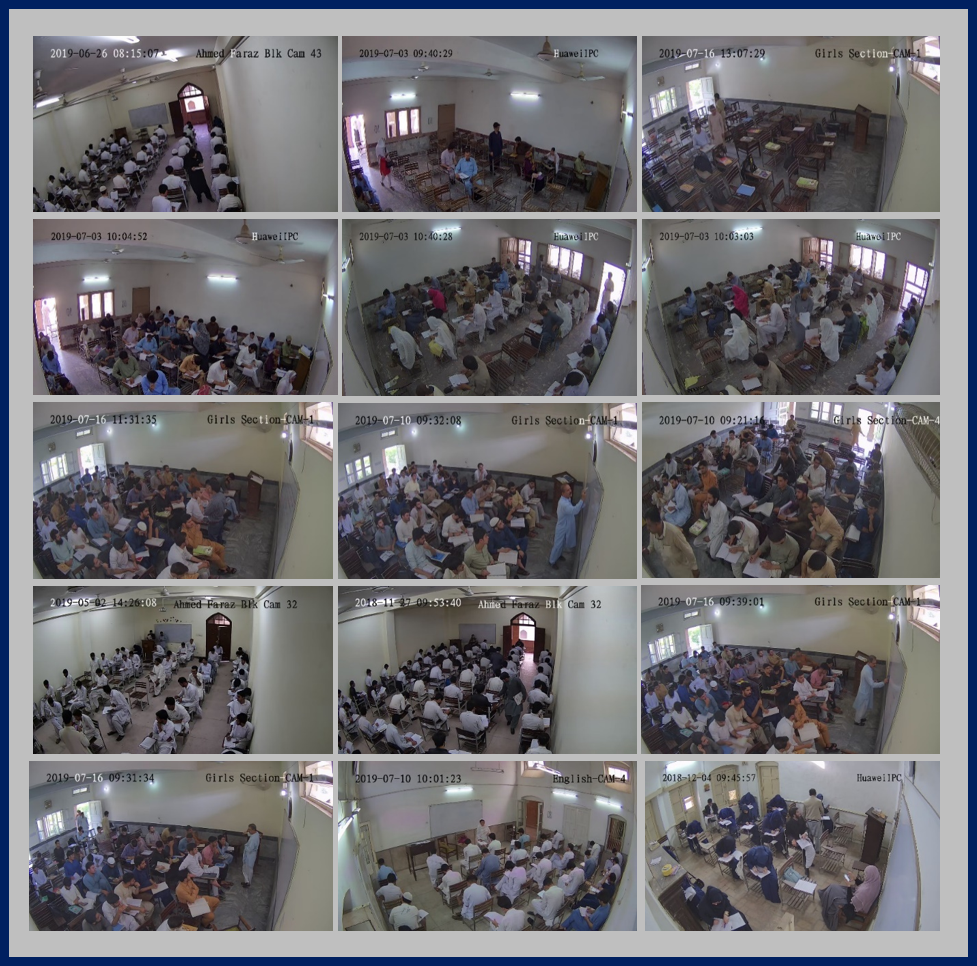

Supplement: Supplemental Information 2 [file peerj-cs-08-911-s002.zip › DataSet/13 - Copy.png]

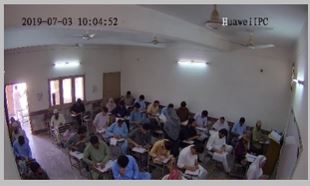

Supplement: Supplemental Information 2 [file peerj-cs-08-911-s002.zip › DataSet/2 - Copy.JPG]

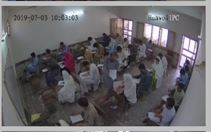

Supplement: Supplemental Information 2 [file peerj-cs-08-911-s002.zip › DataSet/3 - Copy.JPG]

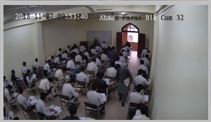

Supplement: Supplemental Information 2 [file peerj-cs-08-911-s002.zip › DataSet/4 - Copy.JPG]

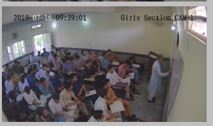

Supplement: Supplemental Information 2 [file peerj-cs-08-911-s002.zip › DataSet/5 - Copy.JPG]

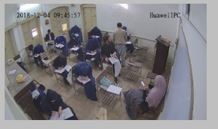

Supplement: Supplemental Information 2 [file peerj-cs-08-911-s002.zip › DataSet/6 - Copy.JPG]

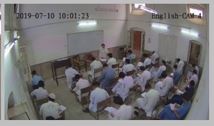

Supplement: Supplemental Information 2 [file peerj-cs-08-911-s002.zip › DataSet/7 - Copy.JPG]

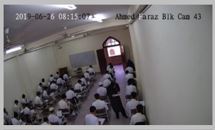

Supplement: Supplemental Information 2 [file peerj-cs-08-911-s002.zip › DataSet/8 - Copy.JPG]

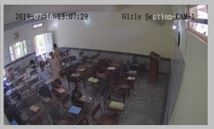

Supplement: Supplemental Information 2 [file peerj-cs-08-911-s002.zip › DataSet/9 - Copy.JPG]
